# Six-month retention and changes in quality of life and substance use from a low-threshold methadone maintenance therapy programme in Durban, South Africa

**DOI:** 10.1186/s13722-020-00186-7

**Published:** 2020-02-21

**Authors:** Andrew Scheibe, Shaun Shelly, Tara Gerardy, Zara von Homeyer, Andrea Schneider, Kalvanya Padayachee, Shalon Balaguru Naidoo, Klaas Mtshweni, Ayanda Matau, Harry Hausler, Monique Marks

**Affiliations:** 1 TB HIV Care, 7th Floor, 11 Adderley Street, Cape Town, South Africa; 2 Urban Futures Centre, Steve Biko Campus, Durban University of Technology, Durban, South Africa; 3 Department of Family Medicine, University of Pretoria, Pretoria, South Africa

**Keywords:** Heroin use, Opioid use, Opioid agonist treatment, Methadone maintenance therapy

## Abstract

**Background:**

Emerging data points to a potential heroin use epidemic in South Africa. Despite this, access to methadone maintenance therapy and other evidence-based treatment options remains negligible. We aimed to assess retention, changes in substance use and quality of life after 6 months on methadone maintenance therapy provided through a low-threshold service in Durban, South Africa.

**Methods:**

We enrolled a cohort of 54 people with an opioid use disorder into the study. We reviewed and described baseline socio-demographic characteristics. Baseline and 6-month substance use was assessed using the World Health Organization’s Alcohol Smoking and Substance Use Involvement Screening Test (ASSIST) and quality of life, using the SF-12. We compared changes at 6 months on methadone to baseline using the Wilcoxon signed rank test and paired-tests for the ASSIST and SF-12 scores, respectively. McNemar’s test was used for comparisons between paired results of categorical variables relating to injecting frequency.

**Results:**

The majority of the participants were young, Black African males, with a history of drug use spanning over 10 years. Retention after 6 months was 81%. After 6 months, the median heroin ASSIST score decreased from 37 to 9 (p < 0.0001) and the cannabis ASSIST score increased from 12.5 to 21 (p = 0.0003). The median mental health composite score of the SF-12 increased from 41.4 to 48.7 (p = 0.0254).

**Conclusions:**

Interim findings suggest high retention, significant reductions in heroin use and improvements in mental health among participants retained on methadone maintenance therapy for 6 months. Further research into longer term outcomes and the reasons contributing to these changes would strengthen recommendations for the scale-up of methadone maintenance therapy in South Africa.

## Background

Opioid use carries significant risks and the resolution of opioid use disorders is challenging [1, 2]. Opioids (including heroin) are responsible for four out of five drug related deaths globally [3]. Injecting drug use significantly increases the chances of developing infections and suffering a variety of health issues, including contracting HIV and hepatitis C (HCV) [4]. Stigma, social exclusion, a lack of accessible services (including healthcare) and criminalisation add to the challenges confronting opioid dependent people [5]. Dependent opioid users experience unpleasant and painful symptoms of physical withdrawal unless they take regular doses (three-six hourly) [6]. Some people who use opioids find temporary relief from the physical and emotional pain they may be experiencing [7–10]. Stopping opioids is often an unrealistic or undesirable outcome for many people who use them. The World Health Organization (WHO) recommends opioid agonist treatment for the treatment of opioid use disorders due to its effectiveness [11].

Methadone and buprenorphine are opioid agonist medications that act on the same receptors in the brain as opioids and relieve symptoms of withdrawal [11]. Opioid agonist treatment involves a trained clinician prescribing agonist medications at appropriate doses. At appropriate doses, this therapy reduces opioid use [11, 12] and the risk of HIV and HCV infection among people who inject drugs, increases adherence to antiretroviral therapy [13] and reduces mortality by up to 75% [14]. Opioid agonist treatment dramatically reduces the medical and societal costs related to opioid use [15, 16]. For every dollar invested in opioid agonist treatment, $5–$12 are saved on potential costs related to opioid use disorders where agonist medications are not prescribed. Additional savings occur when crime and criminal justice costs are considered [13]. Methadone maintenance therapy refers to opioid agonist treatment that is limited to the use of methadone for maintenance.

Community based, low threshold approaches to methadone maintenance improve retention [17]. These enable easy access (including mechanisms to link people with opioid use disorders to treatment), operate with limited resources, and are accessible to participants with limited financial resources and in contexts where drug use is criminalised [17]. Retention is enhanced by offering patient-centred dosing (without ceiling doses), minimising barriers to entry, and adopting approaches that do not demand total abstinence from opioid or other drugs [17–19]. In contrast, high threshold approaches have strict entry requirements, are abstinence-centric, require frequent urinalysis, have rigid dosing, and mandate psychosocial support [17]. Low threshold programmes are therefore a more realistic option for many opioid users [20, 21].

Opioid use is increasing in South Africa with few institutions or mechanisms to prevent what could become a public health crisis [22]. Seizures of heroin in East Africa have risen 100 fold over the past decade, indicating a significant increase in trafficking. Much of the trafficked heroin is bound for South Africa [23]. There is, however, no reliable empirical data on the prevalence of heroin use in the country. The United Nations Office on Drugs and Crime estimates that 0.5% of the population aged 15–64 in South Africa has used opioids in the past year [24], translating to around 184,030 people [25]. Most heroin is smoked or inhaled, either alone or in combination with other substances (e.g. tobacco or cannabis) [26]. The cost of a gram of heroin in South Africa fell threefold from 2004 to 2014 [27]. To date, the country has relied almost exclusively on abstinence based residential treatment or out-patient services with very low ‘success’ rates, usually measured in terms of abstinence [28, 29].

The World Health Organization’s comprehensive package of HIV and viral hepatitis prevention, testing, treatment and care interventions have neither been funded nor implemented by the South African government [30, 31]. South Africa’s first needle and syringe programme started in 2014 in Cape Town, extending to Durban and Pretoria in 2015. In May 2018 the Durban municipality stopped the needle and syringe programme for their cited concerns of insufficient stakeholder consultation and management of used injecting equipment [32], which by January 2020 was still on hold.

Between January and June 2018, 4% of the 7316 people who inject opioids who accessed harm reduction programmes were started on opioid agonist treatment^1^ [33]. Methadone and buprenorphine (± naloxone) are registered for use and only methadone is listed on the essential medicine list for use at hospital level for detoxification (i.e. neither medication is available in the public sector for maintenance) [31, 34]. Agonist medications are prescribed by medical practitioners in the private sector, civil society organisations and universities. Between 2015–2017 South Africa’s consumption of narcotic drugs and buprenorphine was ranked 74th globally (521 defined daily doses per million inhabitants per day; 35 for methadone and 15 for buprenorphine) [35]. The first opioid agonist treatment clinic started in 2011 at Stikland Hospital (Cape Town), with patients self-funding their medications, with a similar clinic starting at Groote Schuur Hospital (Cape Town) 2 years later. The first community-based opioid agonist treatment programme was started in 2014 by a civil society organisation with local government funding. The project provided buprenorphine-naloxone for 3 months to non-injecting opioid users using a high threshold approach with 66% (44/67) completing the project [29]. The limited access to opioid agonist treatment has been partly due to concerns around limited acceptance of the intervention by key stakeholders [31], safety concerns [36], and the impact on the health system and prohibitive costs [28]. Naltrexone in tablet form is registered and only available for use in the private sector. Naloxone is listed on South Africa’s essential medicine list for the management of opioid poisoning across all levels of care [37]. To date, no naloxone distribution programmes have taken place [38].

In response to these issues, the alignment of low-threshold approaches with harm reduction principles [17] and our experience, as well as a donation of methadone, we implemented South Africa’s first low threshold methadone maintenance therapy project. At the time of project planning implementing the project within a government primary care health facility was not possible. Here we present participant baseline social, demographic and drug use data, as well as retention and changes in quality of life and substance use after 6 months on the study. Qualitative research findings around participant experiences will be presented in a separate paper.

## Methods

The study is based on a cohort of 54 people dependent on heroin in Durban, South Africa. The sample size was based on financial and human resources that were available for the project, with a focus on people who smoke heroin (80% of the cohort), reflecting the modes of opioid use in the city [26]. The participants were to receive 18 months of prescribed methadone from a community based low-threshold programme that also provided a needle and syringe and broader harm reduction service in the inner city suburb of Umbilo. Enrolment began in April 2017, and was staggered over 6 months.

Inclusion criteria included: aged 18 years or older; ≥ 12 months history of heroin use with a World Health Organization Alcohol Smoking and Substance Involvement Screening Test (ASSIST) score of ≥ 27^2^ [39]; confirmed recent use of opioids through urinalysis; no pending court case; ability to attend daily clinic visits; a person who could provide support outside the programme; had stable accommodation for the past 3 months; agreed to be contacted for follow-up, and provided informed consent. In an attempt to reduce the risk of overdose and minimise clinical risks for this project people were excluded if they met the diagnostic criteria for an acute alcohol or benzodiazepine use disorder; had a psychotic disorder; or had a history of severe head injury, or cardiac, respiratory or liver condition.

This study was approved by the Institutional Research Ethics Committee of the Durban University of Technology (REC 29/15) and the KwaZulu-Natal Department of Health’s Research Ethics Committee (reference KZ_2016RP14_267). Participants provided written informed consent as part of screening processes. They were not remunerated for participation.

### Stakeholder engagement

Prior to conducting the study, a multi-stakeholder task team was established to inform protocol development and to oversee implementation. This included representatives from the national and provincial Departments of Health and Social Development, police, academics, harm reduction practitioners and (potential) study participants. The task team met bi-annually to reflect on progress, develop opportunities for collaboration, identify referral networks and reflect on challenges and future endeavours.

### Recruitment, pre-screening, screening and initiation

People were informed about methadone prescribing and the programme through organisations providing harm reduction and other health and social services for those who use drugs in the city, as well as through members of the community of people who use drugs. Members of the study team pre-screened potential participants to ensure that they complied with the inclusion criteria stated above.

As part of screening, a study team member collected baseline data on sociodemographic characteristics (sex, age, race, living situation, criminal record) and substance use (using WHO ASSIST and questions around current and previous drug use patterns, including opioid agonists). The study nurse conducted a medical history, physical examination, urine analysis for opioids, and an electrocardiogram (ECG). ECGs were included to identify people with potential cardiac problems at baseline and to gather data on changes over time to generate local safety data. The timing of ECGs was done following the South African guidelines for the management of opioid dependence developed by the South African Addiction Medicine Society [40], which recommends baseline, one month and annual ECG assessment. People with abnormal ECGs were referred to the nearest public hospital for further assessment. A study team member also offered HIV counselling and testing. A multi-disciplinary study team (involving clinical staff, researchers and psychosocial service staff) assessed participants’ suitability and eligible participants were medically assessed by a doctor. Participants signed a treatment contract outlining the nature of the study and the expectations of the participant and staff. The doctor initiated participants onto methadone at an appropriate dose in accordance with the South African Addiction Medicines Society guidelines, which recommends a starting dose of between 10 and 30 mg [41]. The nurse then completed the SF-12 quality of life assessment. This generic preference-weighted health outcomes assessment includes 12 questions. The Quality Metric’s Health Outcomes scoring software enables the generation of quality of life scores [42]. The SF-12 questions around physical functioning, physical state, bodily pain and general health, are used to develop a physical health composite score. Questions about vitality, social functioning, emotional state and mental health are used to develop a mental health composite score. A SF-6D Health Utility Index score is also generated. This preference-based index is used to measure health status, ranging from worst health state (0.0) to perfect health (1.0). The SF-6D score can be used to calculate quality adjusted life years for economic evaluations. The SF-12 has been validated for use in the South African context [42].

### Follow-up visits

Initially, all participants came to the centre for daily observed dosing and were seen by the project nurse and other staff. Participants saw the medical doctor for clinical assessments every 3 to 7 days for the first 2 months as doses were increased and then stabilised, with no ceiling. Participants who had been on a stable maintenance dose for around 3 months were considered for take home doses with monthly medical assessments. A multi-disciplinary team involving the medical doctor, nurse and psychosocial support team decided on the commencement of take home dosing. Take home doses were terminated and daily observed doses reinstated if the participant repeatedly diverted, sold or otherwise misused the methadone supplied. Participants returned to daily observed dosing if their lives had been significantly destabalised. The SF-12, ASSIST, and HIV testing were repeated after 6 months on methadone.

### Psychosocial services

All participants were provided with optional psychosocial support services. Throughout the demonstration project, a social worker and counsellor were available for individual consultations and for group sessions. Following a harm reduction approach, beneficiaries were asked to define their goals and were encouraged to talk without fear of victimisation or judgement of their drug use (past and present). Abstinence was not a requirement for receiving methadone or participation in the study. Shortly after the programme began, beneficiaries started their own peer-led support group. The social worker and/or counsellor assisted in the initial meetings, but fairly quickly the peer-led groups operated independently of ‘professional intervention’. Participation in at least two group sessions and at least one individual session each month was considered regular attendance. A group for family members and other people supporting participants on methadone was established and met every 4 months.

### Multi-disciplinary support

Weekly multi-disciplinary meetings of clinical, psychosocial, support and research staff were held to discuss participants. Clinical and psychosocial plans were developed as required.

### Termination, loss to follow-up and re-entry

Participants who voluntarily terminated their participation were counselled and supported through a down titration process. These participants were viewed as having exited the programme. The protocol allowed for involuntary termination if participants were repeatedly found to divert or traffic methadone. Participants were considered lost to follow-up if they missed 30 consecutive doses. Participants were able and encouraged to restart methadone at any stage if they were deemed lost to follow-up and were interested in re-entry, following a medical and psychosocial assessment.

### Data management

Clinical notes and ASSISTs were completed on paper and captured into a password protected excel database. The SF-12 s were captured into Quality Metric’s Optum PRO CoRE Health Outcomes software (Eden Prairie, Minnesota) and exported into a password protected excel document. Data was merged and imported into Stata v14.2 (College Station, Texas) for analysis.

### Quantitative data analysis

Descriptions of baseline data included proportions for categorical variables and measures of central tendency and dispersion (medians and inter-quartile ranges) for numerical variables. Substance specific and overall substance use scores were obtained from the ASSIST. Mental composite, physical composite and SF-6D scores were outputs from the Optum PRO CoRE Health Outcomes software. Paired t-tests (for normally distributed data) and the Wilcoxon signed rank test (for non-normally distributed data) were used to assess changes in substance use (ASSIST) and quality of life (SF-12 composite scores). McNemar’s test was used to calculate p values for paired data derived around injecting frequency in the preceding 3 months among participants completing baseline and 6-month ASSISTs. Retention in the study was used as a proxy measure for a positive outcome [43, 44].

## Results

### Demographic and social characteristics

**Fig. 1:**
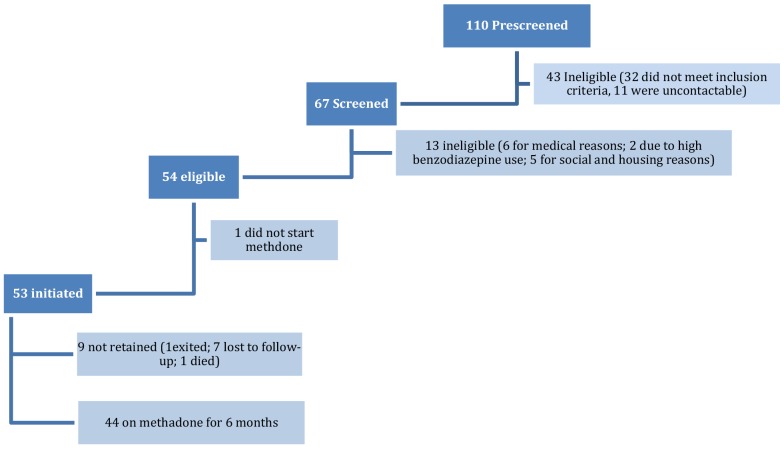
Overview of participant recruitment and enrolment

Recruitment started in August 2017, and of the 110 people pre-screened 61% (67/110) were potentially eligible. Of these, 81% (54/67) signed treatment contracts and were enrolled. We initiated a total of 53 (48% of those pre-screened) people onto methadone (see Fig. 1). All participants who were contacted agreed to be pre-screened.

An overview of sociodemographic and substance use characteristics at baseline is provided in Table 1. The majority of the beneficiaries were young, black African males. Approximately half reported a criminal record and had used drugs for more than 10 years, respectively.

**Table 1:** Characteristics of eligible participants at baseline, eThekwini (n = 54)

Characteristic	N	%
Sex		
Male	51	96
Female	3	4
Age		
Median (IQR)	28	25–32
Race		
Mixed ancestry	3	6%
Black African	34	63%
Indian ancestry	3	6%
White	14	26%
> 10 years drug use history	30	56%
Criminal record	28	53%
Frequency of heroin use (current)		
Daily	1	2%
1–2 times per day	4	7%
3–4 times per day	15	28%
> 4 times	31	57%
Missing	3	6%
Duration of pattern of use		
1–3 months	1	2%
3–6 months	3	6%
> 6 months	47	87%
Missing	3	6%
Injected heroin in last year	10	19%
Drugs detected on urine (baseline)		
Opioids	54	100%
Cannabis	17	32%
Cocaine	8	15%
Amphetamine	1	2%
Methamphetamine	6	11%
Benzodiazepines	1	2%
HIV positive	7	13%
ECG abnormalities^a^	0	0%

^a^No abnormalities suggestive of cardiac disease

### Baseline drug use practices

At baseline, nine participants reported injecting heroin within the previous 3 months while the remainder smoked heroin, usually in combination with tobacco and/ or cannabis. The majority (57%) used heroin more than four times per day. The median baseline ASSIST score for all substances was 81.5 (IQR 67–93), for opioids it was 37 (IQR 33–39) and cannabis 12.5 (IQR 3–23) (see Table 2). Cannabis (32%) was the second most common substance used.

**Table 2:** ASSIST scores at baseline and month 6

Characteristic	Baseline completed (n = 54)		6 months completed (n = 41)		p value
	Median	IQR	Median	IQR	
Substance					
Tobacco	18	15–24	22	18–24	0.4782
Opioids	37	33–39	9	3–23	< 0.0001
Alcohol	0	0–6	2	0–6	0.4388
Cannabis	12.5	3–23	21	17–26	0.0003
Cocaine	0	0–3	6	0–14	0.0541
Amphetamine type stimulant	0	0	0	0	0.1288
Total	81.5	67–93	67	49–90	0.0052
	n	%	n	%	p value
Never injected	41	76%	28	68%	0.1573
Last injected > 3 months ago	4	7%	8	20%	0.0956
Injected < 3 months ago	9	17%	5	12%	0.6547
Injection frequency among those who injected in last 3 months					
< once weekly or < 3 days in a row	3	33%	2	40%	0.5637
> once weekly or > 3 days in a row	6	67%	3	60%	1.0000

### Previous use of opioid agonist medications

In the month preceding initiation, seven (13%) of the participants reported using methadone, with two of them reporting that they stopped due to cost. Among them the median dose was 20 mg (IQR 10–50 mg) and reported dosing frequency ranged from once (n = 4) to thrice daily (n = 1). Additionally, six (11%) people reported using buprenorphine during the month before initiation with a median dose of 6 mg (IQR 4–8 mg) with daily (n = 3) or twice daily (n = 3) dosing.

### Baseline quality of life

The baseline quality of life physical composite score was 55.5 (IQR 47.5–59.3), mental composite score 41.4 (IQR 35–47.3) and SF-6d-R2 0.657 (IQR 0.630–0.738) (see Table 3). The HIV prevalence at baseline was 13% (7/54). No abnormalities were seen on baseline ECGs done on eligible participants.

**Table 3:** Quality of life (SF-12) scores baseline and month 6 (n = 54)

Characteristic	Baseline (n = 50)		6 months (n = 34)		p value
	Median	IQR	Median	IQR	
Physical composite score	55.5	47.5–59.3	55.0	48.2–59.7	0.9153
Mental composite score	41.4	35–47.3	48.7	36.4–51.8	0.0254
SF-6d-R2 (Utility score)	0.657	0.63–0.738	0.681	0.618–0.797	0.1981

## Results at 6 months

### Retention, methadone dosing and psychosocial service uptake

Of the 110 applicants, 53 were initiated on methadone (one client signed consent but did not start methadone), with the median dose of methadone being 30 mg (IQR 30 mg). After 6 months, 81% (44/54) remained in the study. Seven people dropped out and could not be traced and two voluntarily exited. Of the nine people who started methadone and were not retained, 78% (7/9) left within 3 months of initiation. The median methadone dose among participants retained after 6 months was 55 mg (IQR 40–70 mg). The median methadone dose among participants lost to follow-up was 50 mg (IQR 30–60 mg). At the end of the 6-month period, 57% (25/44) were on daily observed therapy at the centre, while the rest received take-home doses. The median number of missed doses among participants remaining on OST after 6 months was 10 (IQR 3–21).

Voluntary group and individual psychosocial services were offered and 74% (40/54) of the participants regularly attended group sessions, 78% (42/54) participated in one or more individual sessions per month with the social worker or counsellor, and 74% (40/54) participated in group and individual sessions.

### Quality of life and health

There was a statistically significant increase in the mental composite score after 6 months (p = 0.0254) among participants retained who completed an SF-12. Changes in the physical composite and SF-6d-R2 scores at 6 months (Table 3) were not significant. No new HIV infections were recorded among beneficiaries retained in the programme at the 6-month time point.

### Drug use

Changes in ASSIST scores between baseline and at 6 months are shown in Table 2. Among those retained, there was a notable reduction in overall substance use. Overall, ASSIST scores decreased from a median of 81.5 to 67 (p = 0.0052). Opioid ASSIST scores reduced significantly from 37 to 9 (p < 0.0001), while cannabis increased significantly from 12.5 to 21 (p = 0.0003). There were insignificant increases in reported use of cocaine, and alcohol at 6 months. Among the 13 people who did not complete the 6 month ASSIST, 9 reported to have never injected at baseline.

### Safety and adverse events

One patient, without a history of injecting drugs, who was living with HIV was lost to follow-up and died as a result of TB infection. One client had an opioid related overdose requiring supportive treatment. Two participants had mild increases in their QTC interval measurements after one month, both of which normalised after dose reduction and counselling.

## Discussion

There are three key findings when comparing 6-month data with baseline from South Africa’s first low-threshold methadone maintenance therapy demonstration project. First, the retention was high (81%). Second, there were significant changes in substance use, specifically a reduction in opioid use and an increase in cannabis use. Third, improved mental health status was identified among retained participants. While the cohort is small and caution should thus be exercised in generalising from this study, these findings are indicative of the value of methadone maintenance therapy in a localised South African setting. Participants’ unanimous acceptance of procedures before initiating methadone points towards the acceptability of the implemented approach. The study is important as it demonstrates the strong positive outcomes of an observed and structured methadone maintenance therapy programme in an upper-middle income country context. Thirteen percent of the beneficiaries had previously accessed and used methadone, but in a non-structured, non-observed manner. The time taken to engage with stakeholders and develop protocols based on local and international recommended practice provided the setting for the study to successfully take place.

### Retention

Retention is considered the best proxy indicator for positive outcomes in the resolution of opioid use disorders [43]. Internationally, retention rates for methadone maintenance therapy differ widely. At 6 months, rates of between 3 and 88% have been identified in the literature [45]. It has been suggested that the benchmark for methadone maintenance therapy is 50% [46]. However, a 2014 review of medication for opioid use disorder treatment programmes in lower and middle income countries reports average retention in methadone programmes as 72% (range 64–80%) at 6 months and 56% (range 46–68%) at 12 months [47].

The results from our study show 6-month retention rates that are at or above retention rates found in similar settings. However, the results are not totally unexpected. The programme was designed to include a number of factors that are predictive of high retention rates such as stable accommodation and a support person. Studies have repeatedly shown that individual characteristics are not predictive of outcomes, and that contextual factors including programmatic and social factors are predictive of positive outcomes. Programmatic predictors of high retention rates include individualised flexible dosing with no ceiling dose [48], accessibility (including cost and geographical position) [49] and therapeutic relationships [50]. Social factors include peer and family support [51]. The potential of take home doses also increases positive social factors [52]. These factors were included in the study design. Most of these features are common to low threshold programmes, which show higher levels of retention than higher threshold services [17–19].

One of the most consistent predictors of retention is methadone dose [53–55]. The globally recommended daily dose range for methadone is 60–120 mg [11]. Interestingly, the median methadone dose among participants retained at 6 months in this study was 55 mg. This could suggest that most beneficiaries did not require very high doses of methadone, most likely because the majority were not injectors [56]. The impact of lower doses was, we believe, mediated by the notable proportion of people on flexible and patient centred dosing (home dosing). Other potential factors include the option for re-initiation after being lost to follow-up and the employment of a non-punitive approach to service delivery.

### Changes in quality of life

Positive change in mental health was significant at the 6-month mark. We infer that this is the result of connections with significant others, including the multi-disciplinary project team, peers and family members. These kinds of connections have been found to reduce feelings of stigmatisation and marginalisation and allow for life normalisation [57, 58]. While the analysis did not indicate any notable change in overall physical health, it is useful to bear in mind that there was low HIV prevalence at baseline, which did not change after 6 months.

### Heroin and other drug use

The notable drop in the overall ASSIST score across all substance classes was to be expected and is supported by decades of research on the effectiveness of methadone [16, 43, 44, 48, 53, 59–64]. There was a significant increase in the use of cannabis amongst participants, however the reasons for this increase was not investigated. While some studies suggest that cannabis use is a predictor of lower retention rates [65, 66], a 2009 review concludes that it is not associated with worse opioid use outcomes. The review notes that there is no need to address cannabis use during methadone maintenance therapy unless the service user requests assistance [67]. A recent longitudinal study confirmed that daily cannabis use was related to better treatment outcomes [68]. Cannabis is a significantly less harmful substance than heroin [2, 69], is cheaper and because of a recent South African Constitutional Court ruling, the use of cannabis in a private space is now legal [70]. The reported levels of alcohol use was lower than expected, considering that alcohol is the most commonly reported substance of use among patients accessing substance use treatment services in the province [71]. An explanation for this could have been under-reporting of alcohol use.

## Limitations

It was difficult to recruit women into the programme, perhaps because of the stigma attached to drug use [72]. Gender aside, the demographics in the cohort were fairly representative of the general population of the city in which the study took place.

The small sample size and opportunistic nature of recruitment limit the generalisability of the findings, although our findings align with global experience. The exclusion of people with unstable housing conditions, significant poly-substance use or people with major medical or psychiatric comorbidities, is likely to have contributed to the high retention rate. Two studies from other South African cities on opioid use treatment services have found a high prevalence (up to 49%) of psychiatric co-morbidity among patients accessing in-patient care in the public sector [73, 74].

Another limitation relates to data. Not all participants completed 6-month ASSISTS or SF-12 s and their inclusion in the analysis may have shifted some of findings, particularly in relation to the (increased) use of drugs, especially heroin, and mental health (worsening or no improvement). After initiation, substance use assessment was based on self-report, which could have led to an under estimate of concurrent use. However, supportive responses were used when participants reported drug use.

Our study did not assess details around participants’ prior access and experience of agonist medications or treatment of their opioid use disorder. As a result, we are unable to assess prior access to evidence-based treatment, and the extent to which opioid agonists were accessed illicitly.

### Future research

This paper focuses on baseline and 6-month data. Data at the 12 month and 18 month intervals are also need to be collated and analysed, particularly in relation to the main findings, i.e. retention, drug use, and mental health. Qualitative research is needed to make sense of the facilitators and barriers to retention, as well as the participants’ health and drug use. It is also important that an analysis be conducted of the outcomes of low threshold opioid agonist treatment using buprenorphine and a cost effectiveness comparison in localised South African settings to inform policy and programming.

## Conclusions

The harm reduction and low-threshold approach used in this project was significant in retaining participants up until the 6-month mark. While a range of other contributing factors should be further explored, our interim findings suggest that this model of methadone maintenance therapy in the South African context is likely to be acceptable to people and has the potential to achieve high retention rates.

The decline in the use of heroin and increased use of cannabis is significant. If harm reduction was a primary goal of this project, it was achieved. At the 6-month mark, this demonstration project resulted in participants reducing potential drug related harms. Combined with the improvement in mental health, this provides good reason for increased access to methadone maintenance therapy to those with an opioid use disorder who wish to normalise their lives. It makes sense, then, for government to investigate the provision of methadone maintenance therapy at the primary health care level in the public sector. A low threshold approach, particularly with regard to the use of flexible dosing (including take home dosing) and non-compulsory psycho-social service uptake, would significantly reduce the cost of such a roll-out.

## Data Availability

The datasets used during the current study are available from the corresponding author upon reasonable request.

## Abbreviations

ASSIST: Alcohol smoking and substance involvement screening test
CI: Confidence interval
HCV: Hepatitis C
IQR: Inter-quartile range
UNODC: United Nations Office on Drugs and Crime
WHO: World Health Organization

## Footnotes

1 Between January and June 2018, 260 people who inject drugs were on OST across five major cities through non-government and not-for-profit services.

2 ASSIST provides a score (overall and disaggregated by substance) reflecting the level of risk (low, moderate and high).
